# Lumbar spinal stenosis associated with peripheral arterial disease: a prospective multicenter observational study

**DOI:** 10.1007/s00776-012-0311-z

**Published:** 2012-09-28

**Authors:** Kazuhide Uesugi, Miho Sekiguchi, Shin-ichi Kikuchi, Masahiro Kanayama, Kazuhisa Takahashi, Kazuhiro Chiba, Minoru Doita, Yasumitsu Toribatake, Hiroshi Matsuo, Kazuo Yonenobu, Yukihiro Matsuyama, Shin-ichi Konno

**Affiliations:** 1 Department of Orthopaedic Surgery, Fukushima Medical University School of Medicine, 1-Hikarigaoka, Fukushima, Fukushima 960-1295 Japan; 2 Spine Center, Hakodate Central General Hospital, Hakodate, Japan; 3 Department of Orthopedic Surgery, Graduate School of Medicine, Chiba University, Chiba, Japan; 4 Department of Orthopedic Surgery, Keio University, Tokyo, Japan; 5 Department of Orthopedic Surgery, Kobe University, Kobe, Japan; 6 Department of Orthopedic Surgery, Koseiren Takaoka Hospital, Takaoka City, Japan; 7 Matsuo Vascular Ultrasound Laboratory, Matsuo Clinic, Osaka, Japan; 8 Department of Orthopedic Surgery, Osaka Minami Medical Center, Osaka, Japan; 9 Department of Orthopedic Surgery, Hamamatsu University School of Medicine, Hamamatsu, Japan

## Abstract

**Background:**

Intermittent claudication is a common symptom of both lumbar spinal stenosis (LSS) and peripheral arterial disease (PAD) in middle-aged and elderly people. However, the prevalence and clinical characteristics of LSS with PAD (LSSPAD) have not been investigated in a multicenter study. The aim of this study was to investigate the prevalence and clinical characteristics of LSS associated with PAD.

**Methods:**

570 patients diagnosed with LSS using a clinical diagnostic support tool and MRI at 64 facilities were enrolled. We evaluated each patient’s medical history, physical findings, ankle brachial index, Japanese Orthopaedic Association Back Pain Evaluation Questionnaire (JOABPEQ) score, and the Short Form 36 (SF-36) score. Statistical analyses were performed to compare LSSPAD patients and LSS patients without PAD using the *t* test, Mann–Whitney’s *U* test, and multivariate recurrence analysis. *p* values of <0.05 were considered statistically significant.

**Results:**

The LSSPAD group comprised 38 patients (6.7 %); 20 (3.5 %) had pre-diagnosised PAD while 18 (3.2 %) had undetected PAD. The clinical characteristics of these patients were advanced age, diabetes, and a history of ischemic heart disease and cerebrovascular disorder. 570 patients enrolled, and 448 (78.6 %) of those patients were followed up at three months after enrollment. Pain in buttocks and legs improved less in the LSSPAD group than in the LSS group (*p* < 0.05). Improvements in the “general health” score in SF-36 were lower in the LSSPAD group than in the LSS group (*p* < 0.05).

**Conclusions:**

Advanced age, diabetes, and a history of cerebrovascular disorder and ischemic heart disease were associated with LSSPAD. Because LSSPAD patients show less improvement in QOL than patients with LSS but without PAD do, clinicians should consider the coexistence of PAD in LSS patients.

## Introduction

Lumbar spinal stenosis (LSS) presents with lower extremity symptoms [1, 2], including neurogenic intermittent claudication as a typical symptom [3]. On the other hand, vascular intermittent claudication is also a typical symptom of peripheral arterial disease (PAD) [4, 5]. Since there is overlap in the ages at which patients develop LSS and PAD, it is important to differentiate the claudication caused by these two different pathologies.

PAD refers to a circulatory disorder caused by stenosis or occlusion of the artery by arteriosclerosis or vasculitis. It encompasses conditions such as arteriosclerosis obliterans (ASO), Buerger’s disease, and acute arterial obstruction. Progression of PAD increases the risk of severe vascular events and even death [6–8]. Early diagnosis and treatment of PAD can improve the hemodynamics of the lower extremities and reduce the risk of fatal or nonfatal cardiovascular events [4, 5]. LSS patients with PAD have been reported [9]; so, when diagnosing LSS, it is important to bear in mind that concurrent PAD is possible. The prevalence of LSS associated with PAD remains unclear because there have been no large-scale epidemiological studies on this issue. One likely reason for this is that there are no established diagnostic criteria for LSS, based on an international consensus. The Japanese Society for Spine Surgery and Related Research, therefore, developed a diagnosis support tool for LSS (sensitivity of 92.8 %, specificity of 72.0 % [10]). Large-scale epidemiological studies of LSS can be executed using this tool.

The measurement of the ankle brachial index (ABI) is recommended for the diagnosis of PAD [5]. The ABI is the ratio of the arm systolic blood pressure (at the brachial artery) to the ankle systolic blood pressure (at the posterior tibial artery or dorsalis pedis artery) [11]. Highly sensitive and specific diagnosis of PAD is possible using the ABI, comparable to that obtained using angiography or Doppler examination [4, 5, 7, 8, 11–14]. Patients with a resting ABI of below 0.9 may have arterial stenosis that affects hemodynamics [4]. In recent years, instruments that automatically measure the ABI have been developed. Using such automatic ABI devices, reliable data can be easily and quickly obtained [6, 16, 17].

As society ages, it is thought that the number of patients with both PAD and LSS will increase. However, the prevalence of PAD in cases of LSS, and the clinical characteristics of such patients, remain unclear. Thus, the aims of the present study were to determine the prevalence of PAD in LSS patients, to clarify the clinical characteristics of patients with concurrent LSS and PAD, and to clarify the treatment course for these patients.

## Methods

### Ethics

This study was approved by the ethics committees of the participating research institutions. Written informed consent was obtained from all patients.

### Study design

This study was a prospective multicenter observational study, conducted under the guidance of the Japanese Society for Spine Surgery and Related Research. The research team consisted of LSSPAD project members. The survey was conducted in 64 hospitals nationwide, all of which had attending spinal surgeons. The recruitment period was one year from October 1, 2008 to September 30, 2009.

### Population

The survey subjects were LSS patients who visited and were examined at the participating hospitals during the survey period. The clinical diagnosis support tool [10] for LSS was used to identify patients with LSS (Table 1). Patients were diagnosed with LSS by a spine specialist if (a) they achieved a total score of ≥7 with the LSS diagnosis support tool, and (b) their neurological findings were consistent with spinal canal stenosis found via MRI at that particular lumbar spinal level. Patients with impaired consciousness, serious complications (heart failure, kidney failure, liver failure, respiratory failure), or psychiatric diseases or symptoms were excluded. Those who were pregnant, were breastfeeding, had myelopathy, had a history of lumbar spine surgery, or were attending for a second opinion were also excluded. To avoid bias among the hospitals, the number of patients enrolled at each hospital was limited to 10.

**Table 1:** Scoring scheme used for the diagnostic support tool for LSS

Item	Score
Age	
<60	0
60–70	1
>70	2
Absence of diabetes mellitus	1
Symptoms	
Intermittent claudication (+)	3
Worse when standing for a while	2
Symptoms improve on bending forward	3
Physical examination	
Symptoms induced by having patients bend forward	−1
Symptoms induced by having patients bend backward	1
Ankle brachial index (ABI) ≥0.9	3
Absence or low response of achilles tendon reflex	1
Straight leg raising test positive	−2

Patients with a total score of ≥7 were considered to have LSS

### Investigations at baseline

At the time of enrollment, the patients were interviewed individually to obtain their medical histories. They were asked about symptoms including the presence of intermittent claudication, exacerbation of symptoms when standing up, and improvement of symptoms when bending forward (lumbar flexion). The severity of symptoms was evaluated using a visual analog scale (VAS: 0–100 mm) for lower back pain, buttock or lower extremity pain, and buttock or lower extremity numbness. In physical examinations, we recorded whether symptoms appeared on lumbar forward or backward flexion, whether the Achilles tendon reflex was diminished, and whether the patient had a positive straight leg raising test. Patients were also asked if they had any comorbidities such as hypertension, diabetes mellitus, dyslipidemia, hyperuricemia and cerebrovascular disorders (stroke, cerebral hemorrhage, or transient cerebral ischemic attack), ischemic heart disease (myocardial infarction, angina pectoris, or coronary revascularization), arrhythmia, and carotid artery disease. Lifestyle questions included history of alcohol intake and smoking. Patients who drank routinely were considered to have a history of alcohol consumption. Patients who were current or past smokers were considered to have a history of smoking. Patients underwent hematological tests.

Quality of life (QOL) was evaluated using the Japanese Orthopaedic Association Back Pain Evaluation Questionnaire (JOABPEQ) [18] and Short Form 36 (SF-36) [19]. The JOABPEQ consists of five subscales and the SF-36 consists of eight subscales. With both tests, a higher score means better maintenance of QOL.

A follow-up survey was conducted three months after enrollment. This survey included symptoms, physical findings, the type of treatment for LSS (conservative or surgical therapies), and QOL (JOABPEQ and SF-36).

### Definition of PAD

PAD was diagnosed by ankle brachial pressure index (ABI). Systolic blood pressure was measured with the patient in a supine position using either BP203RPE III (OMRON Co. Ltd., Tokyo, Japan) or VaSera™ VS-1500E (Fukuda Denshi, Tokyo, Japan). ABI was calculated by dividing the systolic blood pressure of the ankle arteries by the systolic blood pressure of the brachial artery. At the time of enrollment, patients who had already been diagnosed with PAD or patients with ABI ≤0.9 in either leg were diagnosed with PAD [4, 5].

### Statistical analysis

Patients with coexisting PAD and LSS were designated the “LSSPAD group,” and those with LSS but no PAD were denoted the “LSS group.” Using the LSS group as controls, an analysis was conducted to identify the characteristics of the LSSPAD group.

To evaluate the clinical characteristics at the time of enrollment, we analysed and compared (using the *t* test,* χ* ^2^ test, Mann–Whitney *U* test, and multivariate logistic regression analysis) the two groups. *p* values of less than 0.05 were considered significant.

In the survey performed three months after enrollment, the* χ* ^2^ test or Fisher’s exact test were used to investigate differences in symptoms, physical examination findings, and types of treatment for LSS (conservative or surgical therapies). A multiple regression analysis adjusted for age, sex, comorbidities, medical history, and the type of treatment for LSS was performed to evaluate the improvement in the symptoms, JOABPEQ, and SF-36. SPSS for Windows (version 16.0; SPSS Inc., Chicago, IL, USA) was used for statistical analysis. Data are presented as proportions and means (±SD).

## Results

### Description of the sample

**Fig. 1:**
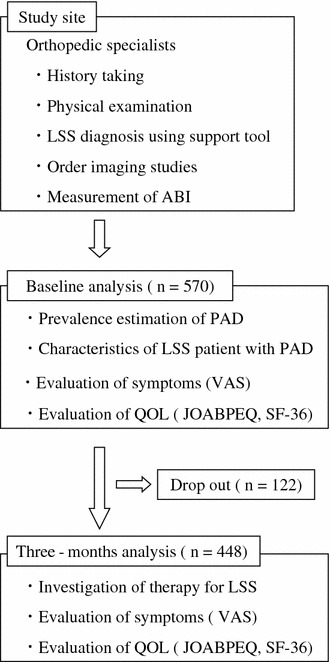
Registration protocol

A total of 570 LSS patients were enrolled (Fig. 1): 303 men and 267 women, with a mean age of 71 ± 8.0 years. Among the 570 LSS patients, 38 (6.7 %) had PAD (LSSPAD group). Of the 38 patients in the LSSPAD group, 20 (3.5 %) had already been diagnosed with PAD prior to enrollment in this study. The remaining 18 patients (3.2 %) had an ABI ≤0.9 and were diagnosed with PAD after enrollment in this study.

### Characteristics of the LSS and LSSPAD patients at baseline and in the survey at three months

The LSSPAD group was significantly older than the LSS group: 75 ± 6.3 years versus 71 ± 8.0 years (*p* < 0.01) (Table 2). Men constituted 71.1 % of the LSSPAD group, a significantly higher percentage than in the LSS group (51.9 %) (*p* < 0.01). VAS scores for numbness in the buttocks or lower extremities were significantly smaller in the LSSPAD group than in the LSS group (*p* < 0.01).

**Table 2:** Characteristics of the LSS and LSSPAD patients at baseline and in the survey at three months

	Mean (SD) or *N* (%)^†^					
	Baseline			Outcome at three months		
	LSSPAD (*n* = 38)	LSS (*n* = 532)	*p*	LSSPAD (*n* = 30)	LSS (*n* = 418)	*p*
Age (%)	75 (6.3)	71 (8.0)	<0.01	75 (6.6)	70 (8.3)	<0.01
Males (%)	27 (71.1)	276 (51.9)	0.02	22 (73.3)	212 (50.7)	0.02
Support tool	11.5 (2.1)	13.2 (2.1)	<0.01	8.5 (4.0)	9.0 (3.8)	0.76
ABI ≤ 0.9	35 (92.1)	–		–	–	
Symptoms						
Presence of Intermittent claudication (%)	36 (100)	483 (92.9)	0.16	16 (57.1)	163 (40.3)	0.08
Worse when standing for a while (%)	35 (94.6)	469 (90.2)	0.56	13 (44.8)	159 (39.4)	0.56
Symptoms improve on bending forward (%)	31 (83.8)	401 (77.1)	0.35	11 (37.9)	141 (34.9)	0.74
Lower back pain (VAS)	47.9 (29.9)	49.3 (28.6)	0.77	39.0 (31.2)	32.4 (27.2)	0.22
Buttock or lower extremity pain (VAS)	53.6 (27.0)	60.1 (28.4)	0.18	42.4 (28.4)	33.9 (31.3)	0.16
Buttock or lower extremity numbness (VAS)	42.3 (32.1)	56.4 (29.9)	<0.01	38.5 (31.1)	36.3 (30.8)	0.71
Physical examination (%)						
Symptoms induced by having patients bend forward	1 (2.7)	20 (3.8)	1.00	1 (3.4)	16 (4.0)	1.00
Symptoms induced by having patients bend backward	20 (54.1)	278 (53.5)	0.94	8 (27.6)	104 (25.9)	0.84
Absence or low response of Achilles tendon reflex	30 (81.1)	349 (67.1)	0.08	19 (65.5)	256 (63.4)	0.82
Straight leg raising test positive	0 (0)	28 (5.4)	0.25	1 (3.6)	5 (1.2)	0.31

*VAS* visual analog scale (scale 0–100 mm)

^†^The total numbers for some items do not add up to the total number in the top row because of missing information

Of the 570 registered patients, 448 completed the follow-up survey at three months—a follow-up rate of 78.6 %. Of these 448 patients, 30 (6.7 %) belonged to the LSSPAD group. The mean age was 75 ± 6.6 years in the LSSPAD group and 70 ± 8.3 years in the LSS group; the mean age was significantly higher in the LSSPAD group (*p* < 0.01) (Table 2). No significant difference in the clinical or physical findings for the LSSPAD and LSS groups was observed.

### Background of patients at baseline

With respect to comorbidities, there was a significantly higher prevalence of diabetes mellitus in the LSSPAD group than in the LSS group (*p* < 0.01) (Table 3). No significant differences were seen between the LSSPAD group and the LSS group in the prevalence of hypertension, dyslipidemia, or hyperuricemia. Regarding the medical history, the LSSPAD group had significantly higher rates of cerebrovascular disorder, ischemic heart disease, and arrhythmia when compared with the LSS group. No significant differences were seen between the LSSPAD group and LSS group in terms of history of alcohol intake or smoking. The LSSPAD group had significantly elevated levels of creatinine compared with the LSS group (*p* < 0.05) (Table 3). No significant difference was seen between the two groups in any other items in the hematological tests.

**Table 3:** Background of patients in baseline

	Mean (SD) or *N* (%)^†^		
	LSSPAD (*n* = 38)	LSS (*n* = 532)	*p*
Comorbidities			
Hypertension (%)	22 (59.5)	240 (45.1)	0.09
Diabetes mellitus (%)	15 (40.5)	103 (19.4)	<0.01
Dyslipidemia (%)	7 (18.9)	81 (15.2)	0.49
Hyperuricemia (%)	3 (8.1)	17 (3.2)	0.13
Past history			
Cerebrovascular disease (%)	8 (21.1)	38 (7.1)	<0.01
Ischemic heart disease (%)	15 (39.5)	32 (6.0)	<0.01
Arhythma (%)	5 (13.2)	19 (3.6)	0.02
Life history			
Drinking history (%)	10 (26.3)	207 (39.6)	0.11
Smoking history (%)	13 (34.2)	161 (30.3)	0.61
Hematological test			
WBC (/mm^3^)	6299 (1508)	6130 (1932)	0.60
Hemoglobin (g/dl)	13.4 (1.7)	13.5 (1.5)	0.73
AST (IU/L)	24.9 (13.9)	24.8 (10.4)	0.95
ALT (IU/L)	21.2 (17.6)	22.4 (14.6)	0.62
BUN (mg/dl)	18.6 (6.8)	16.9 (7.9)	0.18
Creatinine (mg/dl)	0.95 (0.32)	0.78 (0.35)	<0.01
HbA1c (%)	5.9 (1.1)	5.9 (3.9)	0.97
Total cholesterol (mg/dl)	194.7 (32.3)	200.7 (37.9)	0.35
Triglyceride (mg/dl)	137.7 (72.0)	137.5 (75.0)	0.98
LDL-C (mg/dl)	112.3 (28.5)	116.4 (30.9)	0.44
HDL-C (mg/dl)	55.6 (15.3)	58.3 (18.6)	0.40

^†^Total numbers for some items do not add up to the total number in the top row because of missing information

### Multivariate logistic analysis

From the above results, diabetes mellitus, history of cerebrovascular disorder, ischemic heart disease, arrhythmia, high serum creatinine level, and mild numbness in the buttocks or lower extremities were extracted as characteristics of LSS patients with PAD. These extracted factors were then adjusted individually by age and sex (model 1). After this adjustment, factors characteristic to the LSSPAD group (*p* < 0.05) were diabetes mellitus, history of cerebrovascular disorder, ischemic heart disease, and mild numbness in the buttocks or lower extremities. A multivariate logistic regression analysis with a forced entry method was conducted for these factors, including age and sex (model 2).

As a result of multivariate analysis, no sex differences were seen between two groups. The LSSPAD group had a significantly higher proportion of older people, a higher prevalence of diabetes mellitus, and a more frequent history of cerebrovascular disorder or ischemic heart disease than the LSS group (*p* < 0.05) (Table 4). In addition, the average VAS of numbness in the buttocks or lower extremities was significantly smaller in the LSSPAD group than in the LSS group (*p* < 0.05).

**Table 4:** Factors related to LSSPAD in multivariate logistic regression analysis

	Model 1			Model 2		
	Odds ratio	95 % CI	*p*	Odds ratio	95 % CI	*p*
Age	–	–	–	1.06	1.00–1.12	0.04
Sex	–	–	–	1.90	0.85–4.22	0.12
Diabetes mellitus	2.97	1.46–6.04	<0.01	2.63	1.23–5.62	0.01
Cerebrovascular disease	2.67	1.11–6.43	0.03	2.80	1.04–7.55	0.04
Ischemic heart disease	8.00	3.71–17.26	<0.01	7.36	3.30–16.45	<0.01
Arrhythmia	2.66	0.88–8.02	0.08	–	–	–
Creatinine	1.38	0.75–2.52	0.30	–	–	–
Buttock or lower extremity numbness	0.99	0.98–1.00	0.02	0.99	0.97–1.00	0.02

*Model 1*: a multivariate logistic regression analysis adjusted for age and sex,* model 2*: a multivariate logistic regression analysis with a forced entry method (adjusted for age, sex, diabetes mellitus, cerebrovascular disease, ischemic heart disease, and numbness)

### Evaluation of QOL

No significant difference was observed between the LSSPAD group and the LSS group in the JOABPEQ and SF-36 scores at baseline (Table 5).

**Table 5:** JOABPEQ and SF-36 scores at baseline and in the survey performed at three months

	Mean (SD)^†^					
	Baseline			Outcome at three months		
	LSSPAD (*n* = 38)	LSS (*n* = 532)	*p*	LSSPAD (*n* = 30)	LSS (*n* = 418)	*p*
JOABPEQ						
Lower back pain	42.1 (30.8)	47.7 (33.6)	0.32	60.0 (33.5)	67.1 (32.0)	0.26
Lumbar function	64.5 (27.1)	61.6 (29.5)	0.57	63.3 (33.5)	67.5 (28.8)	0.53
Walking ability	27.6 (24.8)	35.4 (27.7)	0.09	41.7 (27.4)	57.5 (31.1)	<0.01
Social life function	34.7 (22.3)	41.8 (22.2)	0.06	42.8 (15.5)	55.3 (24.9)	<0.01
Mental health	43.2 (17.8)	45.8 (18.3)	0.38	52.0 (18.4)	53.7 (19.0)	0.15
SF-36						
Physical functioning	42.4 (23.7)	49.0 (23.3)	0.09	53.6 (21.2)	61.6 (24.5)	0.09
Role physical	45.9 (31.7)	47.9 (28.0)	0.68	42.0 (20.4)	57.3 (28.1)	<0.01
Bodily pain	39.5 (27.1)	34.8 (20.5)	0.19	49.9 (20.5)	51.6 (22.6)	0.69
General health	47.3 (15.1)	47.3 (17.9)	1.00	44.9 (16.8)	52.2 (18.3)	0.04
Vitality	46.2 (22.2)	47.5 (22.2)	0.72	55.2 (20.1)	55.9 (21.6)	0.86
Social functioning	54.9 (28.1)	61.4 (28.7)	0.18	65.9 (22.4)	67.4 (26.7)	0.78
Role emotional	53.8 (33.2)	55.8 (31.2)	0.71	57.5 (26.7)	62.2 (30.4)	0.41
Mental health	54.7 (24.8)	56.8 (22.5)	0.59	64.8 (19.0)	65.9 (21.0)	0.78

^†^Numbers for some items do not add up to the total number in the top row because of some missing information

JOABPEQ consists of 5 subscales. Higher score indicates better QOL

SF-36 consists of 8 subscales. Higher score indicates better QOL

In the JOABPEQ and SF-36 scores at three months after enrollment, the scores for walking ability, social function, role physical, and general health (GH) were significantly lower in the LSSPAD group than in the LSS group (*p* < 0.05).

### Follow-up survey of LSS patients at three months

No significant difference was seen in the types of treatment implemented for LSS between the LSSPAD group (conservative 53.3 %, surgical 46.7 %) and the LSS group (conservative 48.1 %, surgical 51.9 %).

At three months after enrollment, all scores for symptoms, JOABPEQ, and SF-36 showed lower levels of improvement in the LSSPAD group than in the LSS group (Table 6). Based on the results of the multivariate logistic analysis, a multiple regression analysis was conducted, adjusting for age, sex, association of diabetes mellitus, history of cerebrovascular disorder, history of ischemic heart disease, and the types of treatment for LSS. The improvement in the VAS for buttock or lower extremity pain was significantly lower in the LSSPAD group than in the LSS group (*p* < 0.05) (Table 6). No significant difference in the improvement in the JOABPEQ subscales between the LSSPAD group and the LSS group was observed. The SF-36 score showed a significantly lower level of improvement in GH in the LSSPAD group than in the LSS group (*p* < 0.05). No significant difference was seen in the other SF-36 subscales between the LSSPAD group and the LSS group.

**Table 6:** Degrees of improvement and multiple regression analysis of results obtained in the survey performed at three months

	Mean (SD)^†^					
	Total (*n* = 448)	LSSPAD (*n* = 30)	LSS (*n* = 418)	*R* ^2^	*β*	*p*
Symptoms (VAS)						
Lower back pain	−17.0 (33.8)	−9.1 (40.4)	−17.6 (33.3)	0.16	−0.05	0.30
Buttock or lower extremity pain	−25.8 (38.2)	−9.0 (39.7)	−27.0 (37.9)	0.17	−0.12	0.01
Buttock or lower extremity numbness	−19.9 (36.2)	−4.0 (34.2)	−21.0 (36.1)	0.19	−0.09	0.06
JOABPEQ						
Lower back pain	19.6 (37.5)	15.8 (42.1)	20.0 (37.2)	0.08	0.01	0.92
Lumbar function	5.5 (31.4)	−1.5 (31.2)	6.2 (31.2)	0.02	0.07	0.17
Walking ability	22.0 (33.6)	11.8 (32.7)	22.9 (33.5)	0.21	0.07	0.12
Social life function	13.2 (26.4)	5.1 (24.4)	13.9 (26.3)	0.10	0.09	0.08
Mental health	8.1 (19.6)	6.9 (21.2)	8.3 (19.4)	0.11	0.01	0.83
SF-36						
Physical functioning	12.7 (24.6)	11.0 (28.2)	13.0 (24.0)	0.17	−0.02	0.68
Role physical	6.8 (29.9)	−3.0 (32.5)	7.7 (29.6)	0.04	0.10	0.05
Bodily pain	15.9 (26.7)	8.3 (27.4)	16.7 (26.7)	0.11	0.07	0.16
General health	5.0 (17.0)	−1.7 (19.1)	5.8 (17.0)	0.08	0.11	0.04
Vitality	7.9 (22.4)	6.3 (22.7)	8.4 (22.7)	0.10	0.02	0.74
Social functioning	5.6 (29.9)	5.6 (29.2)	5.8 (30.0)	0.02	−0.01	0.82
Role emotional	5.2 (33.7)	3.2 (33.8)	5.6 (33.7)	0.03	0.04	0.49
Mental health	8.3 (23.1)	6.9 (23.8)	8.9 (23.6)	0.07	0.02	0.74

A lower score indicates better condition

JOABPEQ consists of 5 subscales. A higher score indicates better QOL

SF-36 consists of 8 subscales. A higher score indicates better QOL

*VAS* visual analog scale (scale 0–100 mm)

^†^The numbers for some items do not add up to the total number in the top row due to missing data

## Discussion

This is the first nationwide multicenter survey on the prevalence of PAD in patients with LSS in Japan. We found that 6.7 % of the LSS patients had PAD. In other countries, the prevalence of PAD in the general adult population is reported to be 3–19 % [4, 5, 14, 20, 21]. It is also reported that the risk of PAD is significantly higher in older people and in men [12, 14, 22, 23]. In LSS patients, similar to the general population, the risk of concurrent PAD increases significantly with age. However, no sex differences were recognized. Factors other than older age and male sex that are reported to be related to PAD are smoking, hypertension, diabetes mellitus, dyslipidemia, coronary artery disease, and cerebral artery disease [24–27]. We have shown that comorbidity of diabetes mellitus, history of cerebrovascular disorder, and history of ischemic heart disease are characteristic of LSS patients with PAD. Thus, older age, association of diabetes mellitus, history of cerebrovascular disorder, and history of ischemic heart disease may be useful for predicting PAD in LSS patients.

In the clinical setting, the pulse of the dorsalis pedis artery and posterior tibial artery is palpated to examine the peripheral circulation. Patients with diminished femoral artery or posterior tibial artery pulse are at high risk for PAD [28]. Although congenital defects in ankle arteries are rare (dorsalis pedis artery: 1.8 %, posterior tibial artery: 0.18 %) [28], it has been reported that the dorsalis pedis artery cannot be felt in 8.1 % and the posterior tibial artery cannot be felt in 2.9 % of all healthy people [11]. Consequently, the sensitivity of palpation of arterial pulses in the diagnosis of PAD is low [29, 30]. Thus, the absence of arterial pulses in the foot could lead to the overdiagnosis of PAD. The diagnosis of PAD by ABI is noninvasive and simple. Moreover, by setting the ABI cutoff to 0.9, it is possible to screen PAD with high sensitivity and specificity, comparable to that of angiography [15]. In the Trans-Atlantic Inter-Society Consensus (TASC) II treatment guidelines for PAD, screening for PAD with the use of ABI is recommended for all patients with lower extremity symptoms on exertion, patients aged 50–69 with cardiovascular risk factors, and all patients aged ≥70, regardless of risk factors [5]. Many LSS patients with lower extremity symptoms, including intermittent claudication [2, 10, 26], are elderly and at risk for PAD. Therefore, when examining LSS patients, it is important to conduct screening by ABI to avoid overlooking coexisting PAD.

In this study, patients in the LSSPAD group had significantly milder buttock or lower extremity numbness than those without PAD. However, VAS is a subjective evaluation, and it is difficult to use to predict PAD.

No significant differences were seen in the JOABPEQ or SF-36 scores between the two groups at the time of enrollment. Thus, it is difficult to gauge the presence of complicating PAD based on patient QOL or subjective evaluations. In the follow-up survey performed 3 months after enrollment, following adjustment for age, sex, comorbidities, medical history, and whether the patient had undergone surgery, the level of improvement in buttock or lower extremity pain and the GH subscale in SF-36 was significantly lower in the LSSPAD group. The patient’s subjective evaluation of their state of health is reflected in the GH score. If a patient has the impression that their state of health is gradually deteriorating, the score for GH declines [19]. In the LSSPAD group, buttock or lower extremity pain was resistant to treatment, so it is thought that patients’ subjective evaluation of the treatment effect may be lower.

In this study, only about half of the LSS patients with PAD had already been diagnosed with PAD. This means that a large number of LSS patients with PAD had not undergone testing or treatment for PAD. Diagnosing coexisting PAD from claudication or patients’ subjective evaluations is a difficult task, making ABI screening essential in the diagnosis of PAD.

The investigation of the comorbidities and medical histories of LSS patients with PAD in this study was cross-sectional. Therefore, one of this study’s limitations is that the causal relationships between comorbidities, medical history, and coexisting PAD could not be elucidated. Another limitation was that the type of treatment for PAD was not investigated.

In the future, a longitudinal study with detailed classification of each patient’s background will be needed.

## Conclusion

Factors strongly associated with PAD in LSS patients are advanced age, association of diabetes mellitus, history of cerebrovascular disorder, and history of ischemic heart disease. In LSS patients with PAD, buttock or lower extremity pain is intractable, and improvement in QOL is difficult to achieve. When examining patients with LSS, it is necessary to keep PAD in mind.

## References

[1] Thome C, Borm W, Meyer F (2008). Degenerative lumbar spinal stenosis: current strategies in diagnosis and treatment. Dtsch Arztebl Int.

[2] Watters WC 3rd, Baisden J, Gilbert TJ, Kreiner S, Resnick DK, Bono CM, Ghiselli G, Heggeness MH, Mazanec DJ, O’Neill C, Reitman CA, Shaffer WO. Degenerative lumbar spinal stenosis: an evidence-based clinical guideline for the diagnosis and treatment of degenerative lumbar spinal stenosis. Spine J. 2008;8:305–10. 10.1016/j.spinee.2007.10.033 18082461

[3] Takahashi K, Miyazaki T, Takino T, Matsui T, Tomita K (1995). Epidural pressure measurements. Relationship between epidural pressure and posture in patients with lumbar spinal stenosis. Spine.

[4] Hirsch AT, Haskal ZJ, Hertzer NR, Bakal CW, Creager MA, Halperin JL, Hiratzka LF, Murphy WR, Olin JW, Puschett JB, Rosenfield KA, Sacks D, Stanley JC, Taylor LM Jr, White CJ, White J, White RA, Antman EM, Smith SC Jr, Adams CD, Anderson JL, Faxon DP, Fuster V, Gibbons RJ, Hunt SA, Jacobs AK, Nishimura R, Ornato JP, Page RL, Riegel B. ACC/AHA Guidelines for the Management of Patients with Peripheral Arterial Disease (Lower Extremity, Renal, Mesenteric, and Abdominal Aortic): a collaborative report from the American Associations for Vascular Surgery/Society for Vascular Surgery, Society for Cardiovascular Angiography and Interventions, Society for Vascular Medicine and Biology, Society of Interventional Radiology, and the ACC/AHA Task Force on Practice Guidelines (writing committee to develop guidelines for the management of patients with peripheral arterial disease)—summary of recommendations. J Vasc Interv Radiol. 2006;17:1383–97. 10.1097/01.RVI.0000240426.53079.46 16990459

[5] Norgren L, Hiatt WR, Dormandy JA, Nehler MR, Harris KA, Fowkes FGR (2007). Inter-society consensus for the management of peripheral arterial disease (TASC II). J Vasc Surg.

[6] Diehm N, Dick F, Czuprin C, Lawall H, Baumgartner I, Diehm C (2009). Oscillometric measurement of ankle-brachial index in patients with suspected peripheral disease: comparison with Doppler method. Swiss Med Wkly.

[7] Heald CL, Fowkes FGR, Murray GD, Price JF. Risk of mortality and cardiovascular disease associated with the ankle-brachial index: systematic review. Atherosclerosis 2006;189:61–9. 10.1016/j.atherosclerosis.2006.03.011 16620828

[8] Resnick HE, Lindsay RS, McDermott MM, Devereux RB, Jones KL, Fabsitz RR. Howard BV. Relationship of high and low ankle brachial index to all-cause and cardiovascular disease mortality: the Strong Heart Study. Circulation. 2004;109:733–9. 10.1161/01.CIR.0000112642.63927.54 14970108

[9] Dodge LD, Bohlman HH, Rhodes RS (1988). Concurrent lumbar spinal stenosis and peripheral vascular disease. A report of nine patients. Clin Orthop Relat Res.

[10] Konno S, Hayashino Y, Fukuhara S, Kikuchi S, Kaneda K, Seichi A, Chiba K, Satomi K, Nagata K, Kawai S (2007). Development of a clinical diagnosis support tool to identify patients with lumbar spinal stenosis. Eur Spine J.

[11] Khan NA, Rahim SA, Anand SS, Simel DL, Panju A (2006). Does the clinical examination predict lower extremity peripheral arterial disease?. JAMA.

[12] Diehm C, Lange S, Darius H, Pittrow D, von Stritzky B, Tepohl G, Haberl RL, Allenberg JR, Dasch B (2006). Association of low ankle brachial index with high mortality in primary care. Eur Heart J.

[13] Guo X, Li J, Pang W, Zhao M, Luo Y, Sun Y, Hu D (2008). Sensitivity and specificity of ankle-brachial index for detecting angiographic stenosis of peripheral arteries. Circ J.

[14] Ouriel K (2001). Peripheral arterial disease. Lancet.

[15] Bernstein EF, Fronek A (1982). Current status of noninvasive tests in the diagnosis of peripheral arterial disease. Surg Clin North Am.

[16] Beckman JA, Higgins CO, Gerhard-Herman M (2006). Automated oscillometric determination of the ankle-brachial index provides accuracy necessary for office practice. Hypertension.

[17] Benchimol A, Bernard V, Pillois X, Hong NT, Benchimol D, Bonnet J. Validation of a new method of detecting peripheral artery disease by determination of ankle-brachial index using an automatic blood pressure device. Angiology. 2004;55:127–34. 10.1177/000331970405500203 15026866

[18] Mitsuru F, Kazuhiro C, Mamoru K, Shinichi K, Shinichi K, Masabumi M, Atsushi S, Tadashi S, Osamu S, Toshihiko T, Kazuhisa T, Katsushi T, Toshikazu T. JOA back pain evaluation questionnaire: initial report. J Orthop Sci. 2007;12:443–50. 10.1007/s00776-007-1162-x PMC2782112 17909929

[19] Fukuhara S, Suzukamo Y. Manual of SF-36v2 Japanese version. Kyoto: Institute for Health Outcomes & Process Evaluation Reseach; 2004.

[20] Meijer WT, Hoes AW, Rutgers D, Bots ML, Hofman A, Grobbee DE (1998). Peripheral arterial disease in the elderly: the Rotterdam Study. Arterioscler Thromb Vasc Biol.

[21] Selvin E, Erlinger TP (2004). Prevalence of and risk factors for peripheral arterial disease in the United States: results from the National Health and Nutrition Examination Survey, 1999–2000. Circulation.

[22] Alzamora MT, Fores R, Baena-Diez JM, Pera G, Toran P, Sorribes M, Vicheto M, Reina MD, Sancho A, Albaladejo C, Llussa J (2010). The peripheral arterial disease study (PERART/ARTPER): prevalence and risk factors in the general population. BMC Public Health.

[23] Blanes JI, Cairols MA, Marrugat J (2009). Prevalence of peripheral artery disease and its associated risk factors in Spain: The ESTIME Study. Int Angiol.

[24] Hirsch AT, Criqui MH, Treat-Jacobson D, Regensteiner JG, Creager MA, Olin JW, Krook SH, Hunninghake DB, Comerota AJ, Walsh ME, McDermott M (2001). Peripheral arterial disease detection, awareness, and treatment in primary care. JAMA.

[25] Hooi JD, Kester ADM, Stoffers HEJH, Rinkens PELM, van Knottnerus JA, Ree JW (2004). Asymptomatic peripheral arterial occlusive disease predicted cardiovascular morbidity and mortality in a 7-year follow-up study. J Clin Epidemiol.

[26] Kalichman L, Cole R, Kim DH, Li L, Suri P, Guermazi A, Hunter DJ. Spinal stenosis prevalence and association with symptoms: the Framingham Study. Spine J. 2009;9:545–50. 10.1016/j.spinee.2009.03.005 PMC3775665 19398386

[27] Kannel WB, McGee DL (1985). Update on some epidemiologic features of intermittent claudication: the Framingham Study. J Am Geriatr Soc.

[28] Robertson GS, Ristic CD, Bullen BR (1990). The incidence of congenitally absent foot pulses. Ann R Coll Surg Engl.

[29] Hiatt WR, Marshall JA, Baxter J, Sandoval R, Hildebrandt W, Kahn LR, Hamman RF (1990). Diagnostic methods for peripheral arterial disease in the San Luis Valley Diabetes Study. J Clin Epidemiol.

[30] Lundin M, Wiksten JP, Perakyla T, Lindfors O, Savolainen H, Skytta J, Lepantalo M (1999) Distal pulse palpation: is it reliable? World J Surg. 1999;23:252–5. 10.1007/pl00013177 9933695

